# Glutathione and Related Molecules in Parkinsonism

**DOI:** 10.3390/ijms22168689

**Published:** 2021-08-13

**Authors:** Masato Asanuma, Ikuko Miyazaki

**Affiliations:** Department of Medical Neurobiology, Okayama University Graduate School of Medical, Dentistry and Pharmaceutical Sciences, Okayama 700-8558, Japan; miyazaki@cc.okayama-u.ac.jp

**Keywords:** glutathione, neuroprotection, parkinsonism, astrocyte, region specificity, striatum, mesencephalon, oxidative stress, Nrf2, metallothionein, serotonin 5-HT1A receptor

## Abstract

Glutathione (GSH) is the most abundant intrinsic antioxidant in the central nervous system, and its substrate cysteine readily becomes the oxidized dimeric cystine. Since neurons lack a cystine transport system, neuronal GSH synthesis depends on cystine uptake via the cystine/glutamate exchange transporter (xCT), GSH synthesis, and release in/from surrounding astrocytes. Transcription factor nuclear factor erythroid 2-related factor 2 (Nrf2), a detoxifying master transcription factor, is expressed mainly in astrocytes and activates the gene expression of various phase II drug-metabolizing enzymes or antioxidants including GSH-related molecules and metallothionein by binding to the antioxidant response element (ARE) of these genes. Accumulating evidence has shown the involvement of dysfunction of antioxidative molecules including GSH and its related molecules in the pathogenesis of Parkinson’s disease (PD) or parkinsonian models. Furthermore, we found several agents targeting GSH synthesis in the astrocytes that protect nigrostriatal dopaminergic neuronal loss in PD models. In this article, the neuroprotective effects of supplementation and enhancement of GSH and its related molecules in PD pathology are reviewed, along with introducing new experimental findings, especially targeting of the xCT-GSH synthetic system and Nrf2–ARE pathway in astrocytes.

## 1. Introduction

Oxidative stress plays a pathogenic role in neurodegenerative disorders including Parkinson’s disease (PD). Glutathione (GSH) is the most abundant antioxidative molecule in the central nervous system (CNS) and plays a critical role in protecting cells against oxidative stress such as reactive oxygen species (ROS). Previous studies have demonstrated that GSH levels are reduced to approximately 40% of controls in the substantia nigra of PD patients [1,2,3,4]. Since GSH binds to dopamine (DA) quinone (a DA neuron-specific oxidative stress agent) via its thiol group and suppresses DA quinone toxicity, GSH acts as an antioxidant by quenching not only general ROS but also DA quinones [5]. As described below, GSH synthesis in neurons depends on the expression of the cystine uptake system and subsequent GSH synthesis in astrocytes. Thus, GSH synthesized in astrocytes can be regarded as an important neuroprotective molecule, especially in dopaminergic neurons.

Accumulating evidence shows the involvement of dysfunction of antioxidative molecules including GSH and its related molecules in the pathogenesis of PD or parkinsonian models. A number of studies showed neuroprotective effects of supplementation and enhancement of GSH-related antioxidative molecules against neurodegeneration in PD or parkinsonian animals [6]. Furthermore, we found several agents targeting GSH synthesis in the striatal astrocytes that protect nigrostriatal dopaminergic neuronal loss in parkinsonian models [6]. This article contributes an overview of the involvement of GSH and its related molecules in PD pathogenesis, as well as of their disease-modifying properties in PD models.

## 2. GSH and Its Related Molecules in PD Pathogenesis

### 2.1. Role of Astrocytes in GSH Synthesis

GSH is the most potent intrinsic antioxidant against ROS. GSH is a tripeptide comprising the amino acids glutamate, cysteine, and glycine, and it is generated by steps catalyzed by γ-glutamyl cysteine ligase (GCL), followed by GSH synthetase. Cysteine is the rate-limiting precursor for GSH synthesis, and extracellular cysteine is easily auto-oxidized to cystine. Therefore, the cystine-transporting system is essential for supplying cells with the GSH substrate cysteine. However, the cystine transport system is primarily expressed on astrocytes, not on neurons [7,8,9]. Astrocytes take up cystine via the cystine/glutamate exchange transporter (xCT), reduce it to cysteine to synthesize GSH, and consequently release it via a transporter multidrug resistance protein 1 (MRP1) [10]. The released GSH from astrocytes reduces extracellular cystine to cysteine via a thiol/disulfide exchange reaction, and then cysteine can be immediately taken up to neighboring neurons for GSH synthesis [11,12,13]. Thus, GSH synthesis in neurons is dependent on the cystine uptake via xCT and GSH synthesis in surrounding astrocytes. Furthermore, GSH plays an antioxidative role especially in the dopaminergic neurons. Intracellular free DA can be auto-oxidized to DA quinone, which binds to various proteins to form quinoproteins. This quinoprotein formation declines protein function to consequently exert dopaminergic neurotoxicity [14,15,16]. Thus, DA quinones act as dopaminergic neuron-specific oxidative stress agents. Because GSH can bind to DA quinones via its thiol group to competitively prevent binding of DA quinones to functional molecules [14,17,18,19,20], GSH is an important molecule in DA neurons to diminish DA quinone-induced toxicity. These observations indicate that the cystine uptake system and consequent GSH synthesis in astrocytes are essential neuroprotective mechanisms.

### 2.2. Nuclear Factor Erythroid 2-Related Factor 2 Promotes Gene Expression of Antioxidative Molecules

Nuclear factor erythroid 2-related factor 2 (Nrf2), as a detoxifying master transcription factor, activates the expression of various genes, including phase II drug-metabolizing enzymes, such as GSH-related molecules NAD(P)H:quinone oxidoreductase-1 (NQO1) and metallothionein (MT), by binding to the antioxidant response element (ARE) of these genes [21,22]. Nrf2 is sequestered in the cytoplasm by its repressor protein Keap1, and it is constitutively degraded in the ubiquitin-proteasome system in normal conditions [23,24]. When cells are exposed to electrophiles or ROS, Nrf2 is detached from Keap1 via oxidation at cysteine residues of Keap1 and its subsequent conformational changes, and the released Nrf2 translocates into the nucleus to activate various gene expressions [25,26]. Phase II genes enable cellular defense that scavenges ROS, detoxifies electrophiles and xenobiotics, and exerts intracellular antioxidative properties. Recently, Delaidelli et al. demonstrated a robust nuclear expression of phosphorylated Nrf2 in the midbrain and increases in several Nrf2-responsive antioxidative and anti-inflammatory genes in the disease-affected regions in both PD patients and mutant α-synuclein-transgenic PD model mice [27]. Because Nrf2 is expressed mainly in astrocytes, the glial cells can express Nrf2-regulated genes. Indeed, marked increases in the expression of Nrf2-regulating NQO1 in astrocytes and neurons were seen in the substantia nigra of PD [28]. In addition to GSH, MTs are low-molecular-weight and cysteine-rich (20 cysteine residues) proteins that function in metal homeostasis and detoxification through their strong antioxidative, metal-binding, antiapoptotic, and anti-inflammatory properties [29]. The MT family comprises four isoforms. The two most abundant isoforms, MT-1 and MT-2, which are expressed in most organs including the brain, play an important role as antioxidants in neuroprotection in various pathological and inflammatory states to diminish oxidative stress [29,30]. Although MT-1 and MT-2 exert neuroprotective properties, they are not mainly expressed in neurons in the normal or pathological state [31,32]. Numerous studies have demonstrated that MT-1 and MT-2 are produced primarily by astrocytes [33,34,35,36,37,38,39,40,41,42,43,44], and that secreted extracellular MTs from astrocytes mediate neuronal survival and axonal regeneration [31,45]. We found that MT-1/-2 produced and released by/from astrocytes in response to oxidative stress protected dopaminergic neurons against DA-induced neurotoxicity [21]. Furthermore, several drugs, which have an agonistic property on serotonin (5-HT) 1A receptors, protected DA neurons in dopaminergic neurotoxin-induced neuronal death and in PD animal models via astrocytic 5-HT1A receptors, as shown in our previous studies [6,46,47,48]. Astrocytic 5-HT1A stimulation activates the Nrf2–ARE pathway to promote MT expression and its secretion in and from astrocytes, concomitantly enhancing astrocyte proliferation via S100β secretion [48].

Moreover, MT and GSH constitute a redox cycle. When MT reacts with ROS or oxidized GSH (GSSG) to diminish their toxicity, MT releases zinc and forms MT disulfides at the sulfhydryl groups of its cysteine residues, which can be reduced by GSH in the presence of selenium to consequently reconstitute MT with zinc [49]. In other words, MT and GSH reduce each other via their cysteine residues, and cellular reducing molecules such as GSH are necessary to exert the strong antioxidative property of MT.

Thus, GSH-related enzymes and MT, which are Nrf2-regulated low-molecular-weight and cysteine-rich proteins, are expressed at a higher level in astrocytes than in neurons. Several studies, including our previous investigations, have demonstrated that activation of Nrf2 in astrocytes protects dopaminergic neurons against oxidative stress [21,22,48,50,51].

### 2.3. Involvement of GSH and Its Related Antioxidative Molecules in PD Pathogenesis

Various studies have shown the involvement of GSH and its related antioxidative molecules in the pathogenesis of PD patients or parkinsonian animals. Activation of astrocytes has been shown in the animal models of PD using neurotoxins such as 1-methyl-4-phenyl-1,2,3,6-tetrahydropyridine (MPTP), 6-hydroxydopamine (6-OHDA), or rotenone. MPTP is taken up into astrocytes and is converted to neurotoxic 1-methyl-4-phenylpyridinium (MPP^+^), which is taken up into DA neurons via a DA transporter and inhibits mitochondrial complex I to cause specific dopaminergic neuronal degeneration [52]. MPP+ induces apoptosis of astrocytes and increases ROS production and the oxidized GSH (GSSG)/GSH ratio as an indicator of oxidative stress [53]. Another neurotoxin 6-OHDA is also taken up into dopaminergic neurons via DA transporters and then oxidized to produce free radicals, which inhibits mitochondrial complex I [54]. Our previous study demonstrated that astrocytes also express DA transporters [55]. Therefore, 6-OHDA could also be captured by astrocytes, affecting their function. Indeed, 6-OHDA decreased mitochondrial dehydrogenase activity and its membrane potential, augmented ROS level, caspase-3 mRNA level, chromatin condensation, and DNA damage, and induced apoptotic cell death in astrocytes [56]. The depletion of GSH within dopaminergic neurons in the substantia nigra promoted a reduction in mitochondrial complex I activity via increased nitric oxide-related thiol oxidation and age-related dopaminergic neurodegeneration [57]. Aged astrocytes cultured from parkin knockout mice showed impairment of GSH synthesis and failed to protect DA neurons against oxidative stress, proving that parkin deficiency produces abnormal function of astrocytes, leading to vulnerability of dopaminergic neurons to oxidative stress [58]. Innamorato et al. reported that Nrf2 knockout mice showed exacerbated gliosis and dopaminergic neurodegeneration after injections of MPTP [59]. As mentioned above, Nrf2, which induces the expression of GSH-related enzymes, is expressed mainly in astrocytes, and GSH synthesis in neurons requires GSH release from astrocytes. Therefore, Nrf2 knockout mice can exhibit deficiency in GSH synthesis. These observations suggested that a reduction in the GSH-supplying ability of astrocytes results in selective inhibition of mitochondrial complex I and dopaminergic neuronal damage.

### 2.4. GSH Prevents DA Quinone-Induced Neurotoxicity

As mentioned above, reactive DA quinones, which are generated via spontaneous oxidation of cytosolic free DA outside the synaptic vesicle in DA neurons, can covalently react with the sulfhydryl residues on functional proteins, e.g., tyrosine hydroxylase (TH), DA transporter, and parkin, to cause the dysfunction of these proteins by forming quinoproteins in the pathogenesis of PD [14,15,16]. Moreover, it was revealed that DA quinone reacts with α-synuclein, a major component of Lewy bodies or Lewy neurites in PD, to form the DA quinone–α-synuclein adduct which inhibits fibril formation at oligomerization by stabilizing the protofibrils [60]. Therefore, DA quinone has been identified as a dopaminergic neuron-specific pathogenic oxidative stress agent. Our previous study reported that repeated treatment with L-DOPA increased DA turnover and quinoprotein formation, specifically in the striatum of parkinsonian model mice [61,62]. The sulfhydryl groups of free cysteine in GSH and thiol reagents compete with DA or DOPA quinones at the sulfhydryl group on cysteine in functional proteins to prevent the formation of quinoproteins [14,17,18,19,20]. Therefore, GSH acts as an important neuroprotectant, especially in dopaminergic neurons, by quenching not only general ROS but also DA quinones [5].

Lewy pathology including α-synuclein aggregation is one of the hallmarks of PD pathology in the central nervous system (CNS), which appears first in the brainstem and olfactory bulb, before spreading progressively to the substantia nigra to cause dopaminergic neuronal loss and eventually reaching the cerebral cortex [63]. Furthermore, such PD pathology was also detected early in the enteric nervous system (ENS) [64,65]. Nonmotor symptoms including constipation caused by gastrointestinal legions in PD precede motor symptoms by 10–20 years. Therefore, it is hypothesized that α-synuclein fibrils in PD pathology are formed initially in the ENS or sensory nervous system, before spreading in a prion-like fashion from the ENS to the CNS, in part via the vagal nerve [66,67,68,69,70]. Furthermore, bidirectional gut-to-brain and brain-to-gut transmission patterns of α-synuclein have been proposed, and the former pattern depends on gut environmental factors including the microbiota, which elicit mucosal inflammation, oxidative stress, and protein accumulation [71,72,73,74,75]. Because ROS and DA quinones in both CNS and ENS promote α-synuclein aggregation, GSH and its related molecules that diminish oxidative stress would be neuroprotective targets against synucleinopathy.

## 3. Neuroprotective Effects of GSH and Its Related Molecules in PD Pathology

### 3.1. Direct Supplementation of GSH or Its Codrugs

As mentioned above, GSH supplementation would be a plausible approach to protect oxidative stress-induced dopaminergic neurodegeneration. However, direct intravenous chronic injections of relatively high doses of GSH showed no significant ameliorating effects on motor symptoms of PD patients [76], because GSH is unstable and easily degradable to amino acids by peripheral peptidase. Another approach of intranasal GSH treatment was examined [77]. The intranasal treatment of reduced GSH for 3 months improved the unified PD rating scale (UPDRS) total and motor scores in a phase IIb study, although it was not superior to the placebo treatment at the 3 month intervention [78]. Some lipophilic derivatives, conjugated codrugs, or analogues of GSH have been examined in experimental studies. Liposomal GSH, which can cross the blood–brain barrier, spared endogenous GSH and protected paraquat plus maneb-induced neuronal death [79]. Pinnen et al. developed codrugs, which are conjugates of L-DOPA and GSH, as potential treatment for PD. The L-DOPA–GSH codrug may cross the blood–brain barrier, where it is able to induce sustained delivery of DA and restore GSH depletion in the rat striatum [80]. More and Vince synthesized a metabolically stable analogue of GSH as a carrier prodrug for delivery of DA and amantadine [81].

GSH supplementation with a membrane-permeable cysteine precursor *N*-acetylcysteine (NAC), which can cross the blood–brain barrier, exerted neuroprotective effects in dopaminergic neuronal death in parkinsonian models. In the MPTP-treated mouse model of PD, Aoyama et al. found GSH depletion with oxidative stress and dysfunction of a neuronal cysteine transporter excitatory amino acid carrier 1 (EAAC1) [82]. This neuronal toxicity was prevented by pretreatment of NAC. Furthermore, the loss of dopaminergic neurons in the substantia nigra pars compacta with nitrosylation of α-synuclein and microglial activation in EAAC1 knockout mice was reduced by NAC treatment [83]. The role of EAAC1 in neuronal GSH production was well described in the review article of Dr. Aoyama in this special issue [84].

### 3.2. Activation of Nrf2–ARE Pathway or xCT Expression

Extrinsically supplemented GSH or its precursor is easily degradable to amino acids which cannot cross the blood–brain barrier. Therefore, the upregulation of GSH supply from astrocytes is a target for neuroprotection. As described above, activation of the Nrf2–ARE pathway or xCT expression in astrocytes could promote intrinsic GSH synthesis. Overexpression of xCT in astrocytes increased GSH synthesis and supply to protect neurons from oxidative stress [12]. The authors also revealed that synthesis and release of GSH in/from Nrf2-expressing glia would provide experimental neuroprotection [22]. Furthermore, astrocyte-specific overexpression of Nrf2 protects nigral neurons against MPTP neurotoxicity [50]. It was also reported that vulnerability of cortical neurons cultured from Nrf2 knockout mice was dramatically annulled by Nrf2 overexpression in astrocytes [85]. In addition, astrocyte-specific overexpression of Nrf2 delayed motor pathology and synuclein aggregation in the α-synuclein mutant (A53T) transgenic mice [86]. These findings imply that astrocyte-specific modulation of the Nrf2–ARE pathway and xCT is a promising therapeutic target for neuroprotection in PD.

### 3.3. Astrocyte-Targeting Neuroprotective Reagents

**Figure 1:**
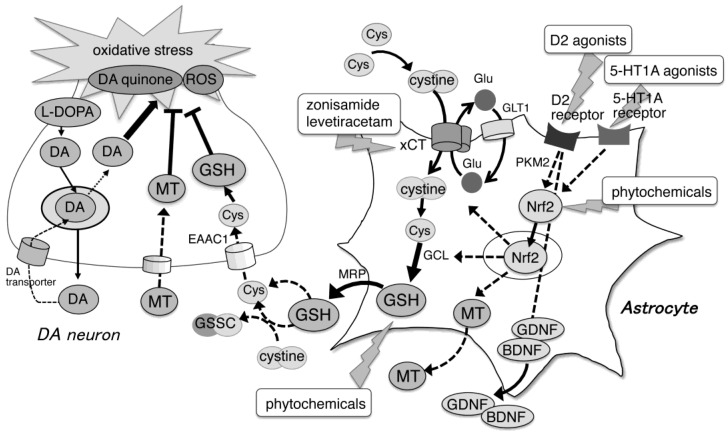
Schematic illustration that summarizes the neuroprotective effects of supplementation and enhancement of GSH and its related molecules in PD pathology.

To date, we have found several astrocyte-targeting neuroprotective drugs and agents, such as DA agonists, antiepileptic agents, and phytochemicals, to increase intrinsic GSH in PD models (Figure 1).

We previously reported that L-DOPA treatment increases L-DOPA uptake into striatal astrocytes and astrocytic GSH release, exerting neuroprotective effects on DA neurons in the presence of astrocytes. These effects were counteracted by concomitant treatment with an L-DOPA metabolite 3-*O*-methyldopa, which is formed by catechol-*O*-methyltransferase (COMT), while they were enhanced by treatment with a COMT inhibitor entacapone [87].

#### 3.3.1. DA Agonists

Some DA agonists that are antiparkinsonian agents were shown to have neuroprotective effects in parkinsonian models [88,89,90]. Pramipexole and ropinirole have been reported to ameliorate dopaminergic neurodegeneration after MPP+ treatment via upregulation of glial cell line-derived neurotrophic factor (GDNF) and brain-derived neurotrophic factor (BDNF) secretion from mesencephalic astrocytes [91]. Furthermore, pramipexole-induced upregulation of BDNF from astrocytes also exerted neuroprotective effects against ubiquitin-proteasome impairment [92], although pramipexole failed to demonstrate an ability to slow the progression of PD in a randomized delayed-start design study using single-photon emission computed tomography (SPECT) [93]. In addition, stimulatory effects of bromocriptine, pergolide, cabergoline, and SKF-38393 were examined on the synthesis and secretion of neurotrophic factors (nerve growth factor, BDNF, and GDNF) in cultured astrocytes [94,95]. Bromocriptine upregulates Nrf2 expression and translocation, as well as activates quinone reductase NQO1, which is increased in the substantia nigra of PD patients [28], to protect neurons against oxidative stress [96]. Our previous studies demonstrated that cabergoline and ropinirole increased the expression of GSH-related enzymes and GSH content to ameliorate the reduction in dopaminergic neurons in a parkinsonian model [97,98,99]. Recently, Wie et al. reported that the astrocytic DA D2 receptor regulates GSH synthesis via pyruvate kinase M2 (PKM2)-mediated Nrf2 transactivation [100]. This implies a possible molecular mechanism underlying the GSH-increasing effects of DA agonists. Furthermore, Shao et al. demonstrated that the astrocytic D2 receptor modulates innate immunity, whereas astrocytic D2 receptor activation suppresses neuroinflammation in the CNS through an αB-crystallin-dependent mechanism, which is known to suppress neuroinflammation [101]. In addition, treatment with selective D2 agonist quinpirole prevented MPTP-induced dopaminergic neurotoxicity in the substantia nigra through partial suppression of inflammation. These studies suggest that the astrocytic D2 receptor would be an important target controlling innate immunity in the CNS to open a new chapter in the therapeutic strategy for PD.

#### 3.3.2. Antiepileptic Drugs

Our previous studies demonstrated that two antiepileptic drugs increased xCT expression and GSH levels in striatal astrocytes and exerted neuroprotective effects against dopaminergic neurodegeneration in parkinsonian mice [102,103]. Zonisamide (1,2-benzisoxazole-3-methanesulfonamide) was originally developed as an antiepileptic drug in Japan, and it is used clinically in many countries. Dr. Murata found that the combination of zonisamide and L-DOPA improved motor impairment in PD patients [104,105,106]. Since 2009, zonisamide has been used as a novel antiparkinsonian therapeutic drug in Japan. We reported for the first time that zonisamide treatment upregulated xCT expression and GSH synthesis in astrocytes and prevented dopaminergic neurodegeneration in parkinsonian mice according to immunohistochemistry analyses and measurements of total GSH content in vitro and in vivo [102]. It was recently reported that zonisamide prevented dopaminergic neuronal loss in lactacystin-injected parkinsonian mice; however, zonisamide failed to increase xCT expression and GSH content in the basal ganglia [107], according to the detection of xCT expression in the striatum or midbrain using Western blot analysis, where only reduced GSH but not total GSH was measured (GSSG + GSH). Levetiracetam has exhibited broad-spectrum effects on partial and generalized seizures in several animal models of epilepsy [108,109,110], and its antiepileptic effects are related to the inhibition of voltage-operated K^+^ currents [111] and N-type Ca^2+^ channels [112]. Unlike other antiepileptic drugs, levetiracetam binds specifically to synaptic vesicle protein 2A (SV2A) [113,114,115], and this is considered to mediate the main antiepileptic action. Experimental studies have demonstrated an upregulation of xCT expression by levetiracetam treatment [103,116]. We demonstrated that levetiracetam upregulates astrocytic xCT expression followed by GSH synthesis, thus acting as a neuroprotectant against progressive dopaminergic neurodegeneration [103]. xCT expression is regulated by the Nrf2–ARE pathway, but Nrf2 was not activated after levetiracetam treatment in our studies. The mechanism of xCT upregulation by levetiracetam remains unclear.

The cystine transporter xCT is a part of the cystine/glutamate antiporter x_c_^−^ system which is composed of the 4F2 heavy chain (4F2hc) and the light chain xCT. This x_c_^−^ system imports cystine into cells in exchange for glutamate. It is supposed that upregulation of xCT may lead to an increase in the extracellular levels of glutamate and cause its excitotoxicity. These findings suggest that xCT would play a detrimental role as a major source of extracellular glutamate in certain pathological conditions, although xCT can provide neuroprotective effects via upregulation of GSH synthesis even in normal conditions. Massie et al. demonstrated neuroprotection against 6-OHDA-induced neurotoxicity in x_c_^−^-deficient mice, suggesting the involvement of x_c_^−^ system activation in the pathogenesis of PD [117]. However, the released glutamate can be taken up via glutamate transporter GLT1 which is specifically expressed in astrocytes. Indeed, zonisamide significantly increased GLT1 expression in astroglial C6 cells in our experiments (personal data). Furthermore, levetiracetam upregulates GLT1 expression in the brain [118]. Therefore, zonisamide and levetiracetam may control extracellular glutamate through their simultaneous upregulating effects on GLT1 in astrocytes, thereby promoting cysteine uptake through xCT.

#### 3.3.3. Phytochemicals

Dietary phytochemicals possess neuroprotective potential in chronic diseases such as cancer, diabetes, and neurodegenerative diseases due to their anti-inflammatory properties [103,119,120,121]. Some beneficial phytochemicals, such as resveratrol, curcumin, quercetin, and sulforaphane, have demonstrated antioxidative properties via activation of the Nrf2–ARE pathway in astrocytes [85,122,123,124,125]. Some dietary phytochemicals can activate the Nrf2–ARE pathway by modifying cysteine residues of Keap1 [126]. We previously demonstrated that a fermented papaya preparation induced Nrf2 nuclear translocation in astrocytes, increased antioxidative molecules, and provided dopaminergic neuroprotection [127]. The fermented papaya preparation increased the nuclear translocation of Nrf2 and increased GSH levels and MT expression in cultured astrocytes. The fermented papaya preparation treatment also ameliorated 6-OHDA-induced dopaminergic neuronal loss in neuron–astrocyte mixed cultures. Glial conditioned medium from fermented papaya preparation-pretreated astrocytes also protected dopaminergic neurons from 6-OHDA-induced neurotoxicity. Furthermore, Nrf2 activation and an increase in GSH levels were observed in the striatum of mice treated with fermented papaya preparation for 2 weeks [127]. Moreover, phytochemicals showed multitarget neuroprotective effects [128]. Curcumin exerts not only antioxidative property via the Nrf2–ARE pathway but also anti-inflammatory and anti-amyloidogenic profiles [129]. l-Theanine, a major free amino acid component of green tea, can across the blood–brain barrier due to a similar chemical structure to glutamate, where it exerts therapeutic effects on various pathological conditions in the CNS [130,131,132,133,134]. We demonstrated that l-theanine protected DA neurons from excess DA-induced neurotoxicity in the presence of astrocytes via upregulation of GSH in astrocytes, and the mechanism of GSH upregulation was clarified using a GSH assay in a cell-free system in which l-theanine could be incorporated into GSH synthesis as a substrate [135]. Furthermore, treatment with a glial conditioned medium from l-theanine-treated astrocytes prevented neurodegeneration of DA neurons by reducing DA quinone formation. Taken together, these findings indicate that l-theanine protects dopaminergic neurons against quinone toxicity by targeting astrocytes. There are some problems, such as solubility, bioavailability, and dosage, in the clinical application of phytochemicals. Therefore, further studies will be required to provide safer and more effective evidence of the neuroprotective effects of phytochemicals.

## 4. Region-Specific Features of GSH-Related Molecules in Astrocytes

In a previous study, we clarified region-specific profiles of astrocytes against 6-OHDA as a dopaminergic oxidative stress agent using cocultures of mesencephalic neurons and mesencephalic or striatal astrocytes [136]. In the cDNA microarray analysis, mRNA expression of GSH-related molecules such as GST, xCT, and multidrug resistance protein 4 (MRP4) was upregulated in the striatal astrocytes. The 6-OHDA treatment markedly increased total GSH content in striatal astrocytes, whereas it showed no changes in mesencephalic astrocyte. Furthermore, the neurotoxin treatment also significantly increased the levels of nuclear Nrf2, GSH, and NQO-1, specifically in striatal astrocytes. Thus, Nrf2-regulating antioxidative molecules such as xCT, GCL, GSH, MRP4, and NQO-1, which are involved in GSH synthesis/export and redox reaction, were upregulated specifically in striatal astrocytes against 6-OHDA. Such a high responsiveness of these molecules in striatal astrocytes could diminish ROS and/or quinone-induced neurotoxicity. The protective features of astrocytes against oxidative stress are more prominent in striatal astrocytes, possibly by secreting humoral factors in striatal astrocytes. In other words, the poor responsiveness of GSH-related molecules in astrocytes might lead to region-specific vulnerability to neurotoxin-induced oxidative stress. Further studies will be required to clarify the mechanism of such region specificity.
